# A qualitative analysis of the documentation of DSM-5 Cultural Formulation Interviews with non-native speaking patients in a Swedish mental health care setting

**DOI:** 10.3389/fpsyt.2024.1298920

**Published:** 2024-02-14

**Authors:** Malin Idar Wallin, Valerie DeMarinis, Lauri Nevonen, Sofie Bäärnhielm

**Affiliations:** ^1^ Center for Psychiatry Research, Department of Clinical Neuroscience, Karolinska Institutet (KI) & Stockholm Health Care Services, Stockholm, Sweden; ^2^ Transcultural Centre, Region Stockholm, Stockholm, Sweden; ^3^ Department of Public Health and Clinical Medicine, Faculty of Medicine, Umeå University, Umeå, Västerbotten, Sweden; ^4^ Division Mental Health Care, Innlandet Hospital Trust, Hamar, Norway; ^5^ Department of Medical Sciences, Faculty of Medicine and Health, Örebro University, Örebro, Sweden; ^6^ Aleris Psychiatry Täby, Stockholm, Sweden

**Keywords:** cultural formulation, cultural psychiatry, clinical assessment, ethnicity and mental health, cultural identity

## Abstract

**Introduction:**

Cultural variety in expressed symptom presentations of mental health problems creates difficulties in transcultural diagnostic assessments. This emphasizes the need of culturally sensitive diagnostic tools like the Cultural Formulation Interview (CFI). Although the CFI is being implemented worldwide there is a lack of studies analyzing what kind of information it provides when used with new patients in routine psychiatric assessments, and how CFI information contributes to diagnostic evaluations. This study aimed to find out what information the CFI questions revealed when used with non-native Swedish speaking patients. We also wanted to understand how the CFI may facilitate identification of psychiatric diagnoses among these patients.

**Materials and methods:**

The CFI was used as part of a routine clinical psychiatric assessment in an outpatient clinic in Sweden. Interpreters were used in the consultations when needed. A qualitative thematic analysis was used to analyze the documented CFI answers from non-native speaking patients.

**Results:**

We found that the CFI information contained contextualized descriptions of dysfunction and current life conditions, as well as expressions of emotions, often described along with somatic terms.

**Discussion:**

Our results indicate that the narrative approach of the CFI, giving contextualized information about distress and functioning, can facilitate clinicians’ identification of psychiatric symptoms when language, psychiatric terms and understandings are not shared between patient and clinician.

## Introduction

Clinical psychiatric assessment is a complex process including identifying psychiatric disorders as a basis for treatment planning. This process requires a means of trustful communication for the patient and, for clinicians, an understanding of the patient’s distress, expectations and hopes. The assessment may be particularly challenging for patients in a migration context, but also important as migration experiences, in particular refugee migration, involves well known risk factors for mental health distress and psychiatric disorders (1–4). Common mental disorders, such as depression and anxiety, are overrepresented among migrants and especially refugees (5–9). Migration status is also a risk factor for psychotic disorders (10, 11), and refugees have a higher risk of psychotic disorders compared to non-refugee migrants, including schizophrenia and other non-affective psychotic disorders (3). A high comorbidity has been found between depression and PTSD (8, 12, 13) and depressive feelings, and disorders are common aspects of refugees’ collective and personal experiences of loss and trauma (14, 15). Depression is globally a major burden of disease, but according to WHO inaccurate assessments and incorrect diagnosis of depressive problems are a significant barrier to effective care for depression (https://www.who.int/news-room/fact-sheets/detail/depression) (16).

Symptoms of depression are expressed and explained differently between cultures (14, 17, 18), sometimes with lower frequency of psychosocial explanations and a higher frequency of somatic or religious ones (17, 19). Many globally common symptoms of depression are not included in current western nosology and are therefore not identified through ordinary clinical instruments (17, 20). The differences in symptom presentation creates difficulties in generalizing western diagnostic nosology across cultures (17, 20, 21) and may cause an impaired recognition, and therefore an underrepresentation, of depression diagnoses (19, 21). The variation in symptom presentation is sometimes associated with alternative explanatory models of illness, which may also affect assessment of the prevalence of mental disorders (21, 22). Differences in explanatory models of illness may influence help-seeking behavior, treatment, adherence and outcome (23, 24). Understanding the patient’s explanatory models of illness is important for a correct interpretation of symptom presentations and the clinicians’ translation of patients’ symptoms into psychiatric diagnostic categories (25, 26).

Variations in expressions of symptoms and explanatory models pose difficulties, particularly in transcultural psychiatric diagnostics (25, 26), as standard instruments may not adequately reflect the experience of psychiatric disorders across cultures (27). The differences may be especially challenging in the cases of newly arrived migrants and refugees (28). The difficulties in transcultural diagnostic assessments increase the risk that mental disorders among migrants and ethnic minorities are not identified or are delayed. There is also a risk that patients are misdiagnosed as clinicians may misinterpret unfamiliar but non-pathological expressions and behaviors as signs of mental disorder. Incorrect diagnoses and poor quality of psychiatric assessment can lead to sub-optimal or even over-treatment, lack of treatment and poor treatment adherence. This, in addition to decreased trust, may imply negative consequences for the therapeutic alliance (29).

Not only cultural varieties in expression and interpretation of distress can pose diagnostic difficulties in multi-cultural care encounters, but also language barriers may result in communication challenges with non-native speakers (30) and complicate psychiatric assessment. Expressing emotions in the patient’s native language makes an important difference in the clinical assessment, and the use of a trained interpreter is therefore important for good communication when the patient and the clinician do not share the same language (31, 32). However, language interpretation is not uncomplicated and there is a risk that the patient-clinician communication is not correctly translated (33). The combination of language and culture challenges sometimes makes good communication in healthcare situations more difficult to establish with migrants and ethnic minorities compared to people belonging to the majority groups of a given society (34).

A global increased risk of distress and psychopathology among migrants, and especially refugees, combined with difficulties in communication are important clinical issues that require careful psychiatric assessments. The communication difficulties have become an important clinical challenge in Sweden as the country has become increasingly more multicultural and the host to many refugees. Currently, 20% out of the Swedish population is first generation migrants, with Syria and Iraq as the two major countries of origin (SCB https://www.scb.se/hitta-statistik/sverige-i-siffror/manniskorna-i-sverige/utrikes-fodda-i-sverige/) (35). To overcome clinical language barriers, the right to use an interpreter in health care situations is statutory in Sweden when the patient and clinician do not share the same language. The use of interpreters is free of charge for the patient and for the health care provider, as the costs are covered by the Swedish healthcare system (36). Although an interpreter contributes to bridging language barriers there is additionally a need for tools that facilitate clinicians’ understanding of cultural variety in expressions and explanatory models of distress in psychiatric diagnostic assessments in Swedish healthcare.

As culture and social context affect the expression and interpretation of symptoms of distress, the growing number of patients with a migrant background around the world, from diverse cultural contexts, increases the importance of including cultural dimensions of value for the clinical encounter and assessment (19, 25, 26, 37, 38). To respond to the diagnostic challenges in transcultural psychiatry and the need to address culture and context in applying psychiatric diagnoses, the psychiatric diagnostic manual DSM-5included the Cultural Formulation Interview and (CFI) (25, 26).

The CFI is a person-centered method, supporting the exploration of cultural and contextual factors in an individualized and non-stereotypic way. The CFI questions cover cultural and contextual factors related to four domains: 1) definition of the problem, 2) perception of cause, context and support, 3) self-coping and past help seeking, and 4) current help seeking. The CFI can be used in the initial assessment of all patients and may be especially helpful in transcultural diagnostic evaluations (25, 26). The 16 questions in the core CFI can, when needed, be supplemented by an informant version and 12 supplementary modules supporting further gathering of information (25, 26).

In addition to supporting the gathering of cultural and contextual information, the CFI may also be helpful for assessing illness severity, understanding cultural barriers between patient and clinician, and increasing commitment and adherence to treatment (25, 26). The CFI does not replace traditional diagnostic tools and skills but is meant to be an additional instrument for mapping relevant cultural and contextual information (39).

The CFI has been evaluated in different cultural contexts and is increasingly being implemented internationally (18, 40–45). The CFI has been found to improve patient-clinician rapport and communication (46, 47), and to be useful in building trust, elicit important contextual information, and support treatment planning (48). Positive results regarding its feasibility, acceptability and clinical utility have been shown in an international DSM-5 field trial (49), and similar positive results have been found in subsequent applications and evaluations (42, 48, 50). An RCT evaluating the CFI’s effect on psychiatric diagnostics among new patients in a Swedish setting, showed a small effect on psychiatric diagnostics with a higher proportion of identified depression diagnosis when using the CFI in the initial psychiatric assessment. The difference was larger among non-native Swedish speaking patients. A larger proportion of differential diagnosis was also found among the non-native speaking patients (44).

Even though there are studies showing that the CFI facilitates communication in healthcare encounters there is research pointing to its shortcomings and the need for further improvement. Some studies found the CFI questions about identity and background sometimes difficult for clinicians as well as for patients to understand (45, 48, 50, 51). The CFI has been criticized for not including social determinants of health, for example social structures, referring to e.g. health systems and services, employment or educational opportunities and housing, as well as factors resulting in racial and ethnic disparities and how they limit access to health care and social services (41, 52).

To refine and improve the CFI, there is a need to understand what information it provides and what information it contributes to psychiatric assessment. To the best of our knowledge, there is a lack of studies analysing what information the CFI provides when used with new patients in routine psychiatric assessments and how it contributes with information affecting diagnostic evaluations. Moreover, there is a paucity of studies evaluating the use of the CFI with non-native speaking patients.

The overall aim of this study is to find out what information the CFI questions revealed when used with non-native Swedish speaking patients as part of routine clinical psychiatric assessment at an outpatient clinic. We also wanted to understand how the CFI may facilitate identification of psychiatric diagnoses among non-native speaking patients.

## Materials and methods

This study is a part of a larger project evaluating the clinical use of the CFI. Data analyzed in this study originate from the CFI conducted in the afore mentioned RCT (44). In the RCT, the CFI’s effect on psychiatric diagnostics was compared between an intervention group, where the CFI was used as the intervention in addition to standard psychiatric procedures, and a control group where only the standardized procedures were used.

### Setting

The study area of the RCT, from which the data for this study originate, is psychiatric outpatient services, serving a multicultural area in western Stockholm. The area has a culturally diverse population with a high proportion of migrants and refugees (approximately 50% of the population are born outside the Nordic countries compared to 22% in the Stockholm region in general). The population’s social situation is strained; in the area the proportions of low-income and unemployment are approximately twice as high as in the Stockholm region overall (53).

### Study procedure

In the RCT, conducted between August 2015 and May 2017, new patients who had not been in contact with psychiatric care over the past two years were included. The CFI was used with patients who were randomized to intervention, using a randomization system with equal likelihood of assignment, in addition to the standard psychiatric diagnostic procedure which was the same for both intervention and control patients. The CFI was conducted by 15 clinicians who were trained in using the CFI;10 psychologists/psychotherapists, 3 psychiatric nurses, 1 counsellor, and 1 mental health auxiliary. Oral and written information, translated into 12 languages, was given to the patients participating in the RCT at the beginning of the consultation: This included participation being voluntary and could be withdrawn at any time without negative consequences. The core CFI was used in the RCT with all 16 questions included. Sometimes, the interview was divided into two consultations due to lack of time. The CFI was used as early as possible in the consultation but not always during the first encounter. The interviews were not recorded and transcribed but documented by the clinicians taking notes during the interview and later included in the patients’ electronic health record. The interviews were not digitally recorded as earlier research noted concerns among new patients about having their consultations recorded. The CFI answers were documented question by question in a narrative way, and usually in a first person form where the text was written as the patient’s direct narrative with the pronoun, “I”. This was in contrast to the rest of the medical records where patient information was described in a third person form using pronouns like, he, she, or the patient Most of the documentation was written as detailed answers to each CFI question, but some were more of a resumé related to each question. The CFI questions were not always documented in order. At the beginning of the study, questions 6-10 were saved for later and documented just before question 16. This was a choice made by the clinicians in the clinical situation. When the study had been in progress for a while, and the clinicians were more used to the CFI, the questions were asked in the intended order.

The routine assessment praxis at the psychiatric clinic including three outpatient units was followed during the RCT, including routine praxis for using an interpreter. At the clinic, an interpreter is used with new patients when needed. The clinicians are trained to work with, and use, interpreters on a daily basis. An interpreter can be requested by the patient, expressed through information included in the referral, or considered necessary by the clinician. When an interpreter is not used the consultation is in Swedish or sometimes the patient’s native language if the clinician speaks this language. An interpreter is used in 5-15% of the consultations at the clinics. Interpreters used in health care in Sweden have a strict protocol to follow regarding translating only and exactly what is said by the patient during the healthcare encounter. The interpreters are provided free of charge for the patient and also for the healthcare clinic. The interpreters used in the healthcare system are legally bound by confidentiality (https://www.kammarkollegiet.se/download/18.27f1fe4c168c1d817515205f/1551777027993/God_tolksed_mars2019.pd) (54). The group of healthcare professionals working at the clinics in this study speak several languages, which sometimes makes the use of an interpreter unnecessary. An interpreter was used with 11 of 47 patients included in this qualitative analysis. When one was used, he/she was instructed to translate verbatim what the patient said during the whole consultation, including the CFI.

Ethical approval for the research project was obtained from the Regional Ethical Review Board in Stockholm (2015/243-31/2).

### Sample

The study sample for this qualitative thematic analysis consists of the 47 non-native Swedish speaking patients included in the RCT. These patients are, based on the information in their electronic health records, identified as being born abroad or in non-native Swedish speaking families.

### Sample characteristics

Most patients’ country of origin was the Middle East (15%) followed by Sweden or other Nordic countries (11%) and other European countries (11%) (**Table 1**). Arabic (6%) and Spanish (6%) were the most common native languages followed by Kurdish (5%), Persian (5%) and Turkish (5%). An interpreter assisted during the consultations of 11 (23.4%) patients and the mean number of years in Sweden, when growing up in another country, was 19.2 (**Table 1**).

**Table 1:** Sociodemographic characteristics of the sample (N=47).

	N47 (%)
Sex:	
Women	27 (57.4)
Men	20 (42.6)
Missing	0 (0)
Age group:	
17-24	8 (17.0)
25-34	11 (23.4)
35-44	9 (19.1)
45-64	19 (40.5)
Missing	0 (0)
Referral diagnosis:	
Suspicion of psychiatric diagnoses	35 (74.5)
Generic problem description	9 (19.1)
No indication	3 (6.4)
Referring agency:	
Voluntary admission	11 (23.4)
Referral from primary care clinics	28 (59.6)
Referral from other caregivers	7 (14.9)
Missing	1 (2.1)
Country of origin:^1^	
Middle East	15 (31.9)
Sweden or other Nordic countries	11 (23.4)
Other European countries	11 (23.4)
Other	9 (19.2)
Missing	1 (2.1)
Native language:	
Arabic	6 (12.8)
Spanish	6 (12.8)
Kurdish	5 (10.6)
Persian	5 (10.6)
Turkish	5 (10.6)
Other	18 (38.3)
Missing	2 (4.3)
Interpreters used:	11 (23.4)
Number of years in Sweden: (when country of origin not Sweden):	M 19.2

^1^Due to birth or upbringing.

### Data and analysis

The data chosen for analysis are the CFI answers from the 47 non-native Swedish speaking patients, as documented by clinicians conducting the CFI interviews. The documented CFI answers were retrieved from the patient’s electronic health records and copied into a document for analysis. The method chosen for analysing the CFI answers is a qualitative thematic analysis (55). We analyzed the material as a whole and not for each CFI question separately. This analysis strategy was chosen since we wanted to find out what kind of information the CFI questions as a whole revealed, and not each question independently. The CFI promotes a reflective dialogue where the patient may add information relating to one question later during the interview. We found that the patients did not always respond to a question directly when asked but later when they were asked another question and had to reflect. The analysis process comprised six stages. First, after an initial immersion of the material, meaning making units were identified in 10 of the documented CFI’s. Second, these meaning units were structured into conversational topics, to reach a structure for further coding of the material, and a preliminary coding scheme was constructed to facilitate further analysis of the whole data set. Third, during the coding of new interviews, the coding scheme was continually developed and revised. Fourth, the categories were grouped into subthemes, and fifth, into themes (55, 56). Sixth, since a qualitative analysis of comprehensive text material can be fragmentized and misinterpretations made, the interpretation of the meaning units of the defined overarching themes was analyzed and the whole material and original text were carefully reviewed once more.

The analysis process was carried out through a multi-coder process. MIW developed preliminary codes, categories and themes which were discussed and revised in association with SB, until the interpretation of the content and categorization of the meaning units were agreed on. The interpretation of the material and themes were then discussed with VDM and after that all authors participated in revisions of the themes and sub-themes. The analysis process was carried out with support from the software program Nvivo12 (2018) (57).

## Results

The analysis of the CFI answers presents information documented in a narrative form in the patients’ health records. From the qualitative thematic analysis, six themes were revealed based on the information from these 47 interviews: *Managing problems and distress*, *Current life conditions*, *Naming distress in a daily life context*, *Making meaning of current problems and distress*, *Sense of belonging*, and *Present concerns in light of the past* (see **Table 2**). The number of references related to each theme are not equal, and therefore we present the themes most frequently occurring first and generally in descending order. As the documentation from the CFI was often written in the first person, therefore the quotes are also often in this form. However, when the documentation was not written in the first person the quotes are not presented in this form. For confidentiality reasons we chose only to present short quotes when reporting the results.

**Table 2:** Presentation of themes and sub-themes with examples of related meaning units.

Themes	Sub-themes	Meaning units
Managing problems and distress *(358 ref)	*Ideas and thoughts about improvement (112 ref)*	*“Medicine, maybe counseling.”*
	*Help-seeking experiences* *(104 ref)*	*“I have never been given a reason.”*
	*Deciding to share or not to share (68 ref)*	*“Doesn’t describe it at all. Can’t put a name to it.”* .
	*Approach to the problem (38 ref)*	*“Have just let the problems be.”*
	*Strategies for managing the problem (36 ref)*	*“I walk, exercise and try to think about something positive.”*
***Current life conditions (267 ref)***	*The problem in relation to others (126 ref)*	*“The family would say that she is misunderstood.”*
	*Current burdensome social factors (113 ref)*	*“All (official) certificates feel like torture.”*
	*Descriptions of the social context (28 ref)*	*“I live with my husband and have for the past five years, and we have a child.”*
***Naming distress in a daily life context (199 ref)***	*Combining somatic and emotional expressions of distress (68 ref)*	*“I am always tired, tired of the pain.”*
	*Limiting consequences in everyday life (56 ref)*	*“Everything is hard to do at home.”*
	*Thoughts and concerns about cognitive functioning (48 ref)*	*“What is most troublesome is that I forget.”*
	*Negative impact on social relations (27 ref)*	*“Anger destroys love relationships?”*
***Making meaning of current problems and distress (75 ref)***	*Contributing family and childhood-related conditions (36 ref)*	*“One reason is sexual abuse as a child.”*
	*Consequences of war experiences (16 ref)*	*“I panic from noises sounding like weapons.”*
	*Stress and work-related contributing factors (11 ref)*	*“I couldn’t study as I had to work. The work was hard, I blame that.”*
	*Recognizing similar problems among family members (12 ref)*	*“I think I inherited it from my dad.”*
***Sense of belonging (108 ref)***	*Reflections on identity (49 ref)*	*“I feel proud that I have a ‘NATIONALITY’ background.”*
	*Religiosity and its meaning (26 ref)*	*“Right now, I am angry at my faith, I am ‘COUNTRY’ orthodox. Think I, as a woman, am drawn to women.”*
	*Manoeuvring through cultural conflicts (21 ref)*	*“‘ETHNICITY’ girls should be well-behaved; I don’t fit into that template.”*
	*Sense of not belonging (12 ref)*	*“It feels like I’m not a part of this world.”*
***Present concerns in light of the past (109 ref)***	*Difficult life experiences (60 ref)*	*“Didn’t want to choose sides, I couldn’t shoot at neighbours, I refused.”*
	*The history of the problem (28 ref)*	*“I don’t know, I have always been like this. Have always done and said things fast without thinking, since I was a child.”*
	*The past social context (21 ref)*	*“I came alone to Sweden in 2012 and lived first in ‘CITY’. I got a residence permit in 2013 and moved to CITY.”*
	*Personal growth (9 ref)*	*“I was shy, ashamed to be different. But not anymore.”*

*The frequency of occurrence of the references (meaning units) for each theme and sub-theme is accounted for through the numbers in the table.

*For confidentiality reasons, names of cities and countries are quoted as ‘CITY’ and ‘COUNTRY’.

### Managing problems and distress

This is the most referenced theme and covers the patients’ descriptions of ways of managing their problems and distress over time. The theme includes information about patients’ approaches to the problem and possible strategies to manage everyday life. Their narratives include information about whether they had shared the problem with others, and if it was a personal choice or due to their perception of being in an environment that did not allow them to talk. The patients also describe earlier help-seeking experiences as well as their thoughts and ideas about what would be helpful in addressing their problem and improving their situation. The theme covers five sub-themes:

#### Ideas and thoughts about improvement

This sub-theme contains many references and gives information about the patients’ ideas and thoughts about how their situation could be improved. These include requests for different kinds of treatment, often medicine or therapy. Other requests are related to assessments of their expressed problems, need to talk to somebody, symptom relief or simply need for help and comfort.

“To talk about my problems, understand where they come from.”

Other ideas about improvement are related to requests for support in everyday life, like somebody who can help with household chores and be company, help with identifying social contexts to meet other people, finding solutions for housing and financial problems, and the need of understanding from colleagues and authorities.

“That somebody will come home and help me when my husband is working, and I am alone and scared. I need somebody who can be with me and go out with me sometimes.”

Some patients talk about how their own life adjustments or understanding of their own problems might be helpful. Some patients express an uncertainty of what would be helpful, or thoughts related to visions of the future and an altered situation.

“I daydream a lot. Among other things, in my head I build onto my house.”

#### Help-seeking experiences

This second largest sub-theme is also rich in information and describes the patients’ earlier help-seeking experiences, including information about previous healthcare interventions. There are also descriptions of help-seeking experiences outside the healthcare system.

“I have sought help mostly from God. I go to God to get strength to get me through difficulties. To listen to hymns gives an inner peace.”

The patients often convey disappointment related to earlier healthcare interactions, mostly related to a lack of understanding and communication problems. The feelings of lack of understanding are sometimes linked to when the patients did not understand the purpose of the health care intervention. Also, and more frequently, experiences of not being understood are described. One patient expresses this by saying:

“I am surprised that you understand what I say.”

This sub-theme also includes perceptions of culture’s impact on help seeking and healthcare encounters as well as the meaning of identity in treatment and care. Some patients described cultural differences as a problem in the healthcare encounter.

“Doctor and patient have to come from the same culture to understand each other in depth.”

Other patients thought of cultural differences as an asset in a healthcare meeting. Some patients express a contradiction between the prescribed recommendations and their identity.

“I take my medicine but I’m not a medicine person.”

#### Deciding to share or not to share

Through the interviews, the patients convey information about sharing, or not sharing, their problems and distress with others. There is also information about with whom and how they share their problems, if they do.

“I describe the problem to others as tiredness and I blame the pain in my body.”

If they do not talk about their problems the patients reveal if the reason for this is that they do not want to, because of shame or other reasons, if they lack a social context, or if they perceive the latter as not allowing them to share these kinds of feelings and distress.

“Have been thinking that this should be a secret and that I have to manage it.”

#### Approach to the problem

In this sub-theme, the patients describe their approach to the problem. The approach is often of a passive character but feelings of shame or neglecting the problem are also represented. The patients convey absence of earlier help-seeking behavior and sometimes a passive, disregarding or isolating approach.

“I tried to solve the problems on my own and postponed seeking help. Didn’t see it as something you could seek help for.”

Some patients describe an approach of keeping up appearances. The CFI documentation reveals patients’ feelings of shame, leading to distancing oneself from the family, “managing on your own” and expressions of difficulties in managing the situation. Some patients describe the impact of cultural traditions on how they manage their problems, frequently related to stigma.

“In our culture it is not possible to say that I see a psychologist, it means I’m crazy.”

#### Strategies for managing the problem

In this sub-theme, the patients shared different prior and current strategies used to manage their distress. These are sometimes strategies of trying to keep calm and of distraction or avoidance.

“I guess I have escaped (referring to the problem) by shopping or doing laundry or cleaning.”

Some patients describe helpful activities, such as exercise and physical strategies like breathing exercises. There are also strategies of seeking knowledge through internet and media. Some patients follow specific cognitive strategies or routines.

“For example, I turn the paper that you gave me upside down, so that I can listen to you.”

### Current life conditions

This theme also has many references. Here the patients describe current life conditions that they considered to be important for their perceived distress. They describe their social context, the situation of social support and others’ views of the problem, as well as current factors in life that are especially burdensome. The theme covers three sub-themes:

#### The problem in relation to others

This rich in content sub-theme gives information about the patients’ problem in relation to others, including several aspects, such as others’ approach to, and view of, the problem as well as others’ views of what is causing the problem and what would be helpful. The patients express the perception of existing support from people around them and how others relate to their distress. Examples of descriptions of other people’s explanations as to what is causing the problems and distress are:

“Family and friends say that I have worked too hard, that I have worn out my body.”

Others’ views of what could be helpful are described and some patients explain how they are sometimes compared to others who do not suffer from distress.

#### Current burdensome social factors

This sub-theme covers a lot of information about social factors perceived as burdensome in the current situation. Stressful economic and working conditions are described here. The sub-theme also includes perceived strains related to contacts with authorities.

“I feel ´labeled´ by the Swedish Insurance Agency. The feeling is that they want to get rid of you.”

This sub-theme also covers burdensome factors related to migration or refugee status, such as uncertainty of getting a residence permit, difficulties to work, missing the country of origin, language difficulties and feelings of being a stranger.

“My big problem is the fear of what would happen to me and my son if my husband was deported. Sometimes it feels like I will die.”

Stressful social structures are described, such as experiences of racism and discrimination in working situations and in everyday life. Stressful situations caused by challenges in contacts with the Swedish welfare system are also described. Information about stressful family situations such as conflicts or sickness in the family is also included here. There are some descriptions of stressful contextual conditions, such as burdensome living situations. This sub-theme also includes worries about one’s own health.

#### Descriptions of the social context

This sub-theme includes information about the patients’ social context, such as relationships, living conditions and working or school situation. The patients explain their relations to their partner, friends, and family.

“I don’t have that many friends, but I have one good friend. Before I had more friends when I had money.”

### Naming distress in a daily life context

The theme, *Naming distress in a daily life context*, provides information about how the patients convey their distress in terms of its effect on everyday life and social relations. This is about how the patients put emotions describing distress into words, and the way they express thoughts and concerns about cognitive functioning related to the problem. The theme covers four sub-themes:

#### Combining somatic and emotional expressions of distress

This sub-theme focuses on how patients combine emotional and somatic expressions related to their perceived distress. Words describing emotional and somatic experiences were often used together and related to the situation where discomfort arose.

“I close my eyes and it feels like I will die. I have a lump in my stomach, my heart beats, it flashes before my eyes and sometimes my left arm is numb.”

#### Limiting consequences for everyday life

This sub-theme describes how different parts of the patients’ everyday life are affected by the perceived distress, such as an inability to orient or be active, to manage household chores and household finances.

“I do not dare to do things right now. I am at home.”

There is also information on sleeping difficulties and how tiredness and passivity have negative consequences for the patients’ school or working situation. The sub-theme also covers problematic behaviors related to the problem, such as aggressive, or compulsive behaviors, affecting the patients’ everyday life.

#### Thoughts and concerns about cognitive functioning

This sub-theme describes thoughts and concerns about cognitive functioning related to perceived distress. The thoughts can be of compulsive character, but there are also worrying or suicidal thoughts or thoughts and memories of traumatic events. The concerns about cognitive functioning are often related to concentration difficulties and memory loss.

“I have a problem with concentration, worry takes over my mind.”

The sub-theme also includes descriptions of dysfunction. The problems are sometimes, but not often, described in psychiatric terms.

#### Negative impact on social relations

This sub-theme describes how the patients’ distress has a negative impact on social relations, like fear or discomfort in social situations. Other descriptions are related to conflicts or misunderstandings that arise, how the distress or problems have a negative impact on school or working relations. There are also descriptions of how the problems have a negative impact on relationships with family members and friends as well as in love relationships.

“I don’t feel well, and I don’t feel a good enough mother when I am stressed.”

### Making meaning of current problems and distress

In the theme, *Making meaning of current problems and distress*, the patients describe their view of what has contributed to perceived distress and how they explain and make meaning of their current problems. Many patients consider childhood- or war-related difficult events or conditions as contributing factors to their ill health. Several patients have explanations of physically hard or stressful working conditions as contributing factors, and some describe how their family members suffer from similar distress. Only a few explained their problem to be related to psychiatric disorders or conditions, like ADHD. Some considered issues such as denial of one’s own needs, identity confusion or somatic problems as causes. The theme covers four sub-themes:

#### Contributing family- and childhood-related conditions

The documentation from the interviews frequently reveals stories about family- or childhood- related conditions perceived by patients as contributing to current distress. These conditions are often related to upbringing circumstances or abusive events during childhood.

“Maybe feel often that other people lied to me. When I was younger, I was too kind. The other children bullied me because of it. Something broke inside me and I can’t take it any longer.”

Sometimes the contributing conditions are connected with more recently occurring events related to family relations or death in the family.

#### Consequences of war experiences

Some patients explain their current distress through experiences of war. This subtheme includes information about when the patients clearly consider war as a cause of their current problems and distress.

“Half of my family is gone because of the war … They beheaded my brother that I always hung with. It is heavy to carry.”

#### Stress and work-related contributing factors

Descriptions of previous or current working conditions and stresses are re-occurring explanations of current distress. One example of difficult working conditions earlier in life is:

“I have been working since I was twelve years old. In a factory. I lifted very heavy objects. I blame that and stress. I worked in the factory for fifteen years day and night.”

Other work-related contributing factors have to do with a psychological negative impact from recent or current working situations, like stress, discrimination or bullying.

#### Recognizing similar problems among family members

In this sub-theme the patients report that family members suffer from similar distress and how they recognize their own problems among these family members.

“I see that others in the family do the same. I can understand in different ways that they have the same problem.”

They express thoughts about heredity and sometimes relate the problems to culture and traditions.

### Sense of belonging

In the theme, *Sense of belonging*, the patients express their feelings of belonging and groups they connect and identify with. Patients reflect on their identity and sometimes emphasize their identity of origin and their relationship to a Swedish identity. They also share experiences of alienation and their feelings of not belonging, in what they call the Swedish “world”, their “world of origin” or in any “world”. The patients reflect on their experiences of religiosity and its meaning and on cultural conflicts and traditions. The theme covers four sub-themes:

#### Reflections on identity

This sub-theme highlights the patients’ views on their own identity, often an identification with their own culture of origin or of being a foreigner.

“Culturally we are always foreigners. So, we know what we belong to, who we are.”

The meaning of language related to identity is also reflected in the complexity of identity, often in relation to a Swedish identity.

“I try to identify myself with being Swedish. Think that I am born here, try to forget the past. I don’t like the religion, traditions, and politics from my home country. They are not close to my identity.”

Other important identities, like deaf identity or sexual orientation are also included.

#### Religiosity and its meaning

This sub-theme reflects the patients’ experiences of, and reflections on, religiosity and its meaning. Often these are reflections on one’s own relationship to religiosity.

“I believe in God, as a catholic. Don’t go to church often, but God is everywhere. I don’t know if that has affected anything, but it is something positive.”

Sometimes the reflections revolve around bad experiences of actions in the name of religiosity. There are also reflections on how religious beliefs sometimes change over time.

“I have changed my perception about religion, I dare to think more freely.”

#### Manoeuvring through cultural conflicts

In this sub-theme the patients reflect on unwritten rules and traditions of their own cultures of origin. Often the reflections on cultural traditions are in relation to the Swedish culture.

“At home it is different, there are other unwritten rules than in a traditional Swedish family.”

Mostly, there are neutral reflections and experiences of adjusting or not to rules and traditions, but sometimes there is a valuation in the patient’s statement on cultural traditions.

#### Sense of not belonging

In this less frequent occurring sub-theme the patients share their experiences and feelings of being left out and of alienation. They express their emotions of neither belonging in the Swedish ‘world’ nor also in their culture of origin.

“Have always felt left out. It’s two worlds; our world and the Swedish world. Feels like exclusion. I am a ‘svartskalle’* in Sweden and Swedish in my home country.”

### *An abusive slang term

### Present concerns in the light of the past

In this theme, the patients look back and reflect on their lives and backgrounds and talk about present concerns in the light of the past. They share difficult life experiences and describe how the current distress has developed over time. The theme also covers the patients’ perceptions of personal growth over time. It covers four sub-themes:

#### Difficult life experiences

The patients share their stories about difficult events in life. However, these events are not expressed as causing the problem but rather as life-framing experiences and important information from the patient’s past. The difficult life events or conditions are often related to family or upbringing.

“The ‘old man’ hit me, it was a part of my upbringing. But I don’t dislike him because of it.”

Difficult experiences of war and migration are also frequently occurring. These experiences often include traumatic events, like imprisonment, armed conflicts, and separation from family members.

“You Swedes have seen war on TV, while I have been standing there with the choice of shooting or be shot. I have shot to survive.”

#### The history of the problem

In this sub-theme, the patients describe how their problems have developed over time and relate their current situation to life before the current distress. Some patients report that their problems have been there since childhood.

“I think it’s uncomfortable to look people in the eyes. Have been like that since I was a child.”

The patients reflect on how their lives, their identities and their abilities have changed in relation to the problem.

“Yes, it is a crisis now. Before I used to be a good person with education and now, I am zero.”

#### The past social context

This sub-theme contains narratives about the past social context including information about migration and where the patient was born and raised.

“I was born in ‘COUNTRY’, I came to Sweden when I was 13 years old. My dad had gotten a job in Sweden, and we were poor in ‘COUNTRY’.”

There are also descriptions of the family structure in the patients’ upbringing context.

#### Personal growth

This sub-theme is small but illustrates the patients’ perceptions of having undergone personal growth over time in different ways.

These are sometimes illustrations of improvement of symptoms and abilities.

“Now everything is like it used to be. I can hug my children and I don’t feel uncomfortable doing it. I have been able to accept what happened.”

Some patients describe development of their self-image and acceptance of their identity or problems.

“I can’t change my background, my name or what I look like. It has given me uncertainty, but now I am good as I am.”

## Discussion

### Main findings

The study aimed to find out what information the CFI questions revealed when used with non-native Swedish speaking patients, as a part of routine clinical psychiatric assessment at an outpatient clinic. We also wanted to understand how the CFI may facilitate identification of psychiatric diagnoses among non-native speaking patients. The information gathered was documented in patients’ health records and analyzed with the qualitative method of thematic analysis. The documentation of the patients’ responses to the CFI questions by clinicians, was done in a narrative style, mostly using the first person, directly conveying the patient’s expressions. The narrative form often had a lifeline perspective and permeated the material. Our analysis involved an amalgamation of 47 patients’ individual and unique narratives and resulted in identifying six overarching themes: *Managing problems and distress*, *Current life conditions*, *Naming distress in a daily life context*, *Making meaning of current problems and distress*, *Sense of belonging*, and *Present concerns in light of the past*. The overarching themes reflect how the CFI in particular stimulated the sharing of information related to problem description and problem management as well as the patients’ present life situation.

#### Findings on managing distress

The most frequently occurring theme, *Managing problems and distress*, includes descriptions of earlier help-seeking experiences and patients’ thoughts about what could be helpful in the present situation. Information about misunderstandings in previous healthcare encounters and the impact of cultural dimensions on patient – clinician relationships and help-seeking behaviors are also given. It is possible that the time and space provided for talking about these experiences through the CFI process contributed to patients’ feelings of being understood and listened to. The benefits of the interview process not only lie in the information gained that might be helpful in the diagnostic process, but also the patients’ appreciation of the process and feelings of finally being understood. This in turn helped to build trust and improve the patient – clinician alliance, which has been recognized in other studies (48, 50). The trust and alliance-building element of a genuine interest in the patients sharing previous help-seeking experiences and thoughts on treatment can positively affect the trajectory of the current treatment process. This element is of special importance when previous experiences have been perceived as negative and resulting from the clinician’s lack of interest in, or respect for, the patient’s narrative. Understanding the consequences of not including such a trust and alliance-building element into the treatment process adds an important dimension to previous research, recognizing that the clinicians’ unexplored preconceptions about a patient may affect the patient – clinician relationship, particularly in the context of cross-cultural encounters (58).

#### Findings on contextualized information

In our study, the CFI uncovered many contextual descriptions of the patients’ dysfunctions and distress which correlate with some results from other studies, one where care providers reported that the CFI gave information about the patients’ social support network (48). In another study, where the OCF (Outline for Cultural Formulation, a precursor of the CFI) was used, the results showed that it was helpful in giving contextualized information affecting diagnostic categorization (39). In our study, we found patients’ descriptions of their problems as presented in a daily life context of current life conditions and included information about how the problems have limiting consequences on everyday life. Words of emotions were often combined with descriptions of somatic concerns and distress.

Contextualized information related to the patients’ social contexts also continuously emerge through the CFI answers, as well as the negative impact of the problem on social relations. The CFI in its current form has been criticized for not sufficiently eliciting social predicaments and social structures (41, 52). However, for patients in our study, the CFI contributed with rich information about social determinants of health of importance to the patient. For example, the sub-theme, *Current burdensome social factors*, highlights social structures of importance, for example, a strained economy creating problems in everyday life and negatively affecting mental health. Other examples are difficult living conditions, such as having only temporary housing, and experiences of discrimination, as well as other burdens or uncertainties related to migration status. In the latest version of the psychiatric diagnostic manual, DSM-5 TR (26), the CFI text about stressors has been developed. Added to the instructions is text defining stressors as including social determinants of the individual’s mental health. This study took place before these instructions were added. However, our analysis shows that information about social determinants of the individual’s mental health was gathered in accordance with how the questions in the CFI are formulated.

#### Findings on explanatory models and the problems in the light of the past

The CFI questions uncovered information about patients’ explanations of perceived distress, as well as those of the patient’s family or social network. Since the patient’s explanatory models influence help-seeking behavior, treatment, adherence, and outcome this type of information is of importance for treatment planning and care (23, 59). The information about patients’ explanations of distress complies with the DSM-5 attention to explanatory models as key aspects of an individual’s clinical presentation and understanding of distress (25, 26). The CFI documentation also includes narrative descriptions of the patient’s past context and experiences, framing their present situation and current distress. These are not here expressed as causes or explanations of the present concerns by the patients but can be seen as a part of the process of making meaning of distress.

#### Findings on identity and sense of belonging

We found great variation in information related to background and identity and have abstracted this information with the theme “sense of belonging”. This includes experiences of not belonging or feelings of exclusion or being split between the culture of origin and the Swedish culture. The sense of belonging, or lack of belonging, is sometimes described as a burden and sometimes as a resilient factor, and often both experiences are revealed in a patient’s narrative. The CFI questions about cultural identity has been problematized in recent research (45, 48, 50, 51) and identity as a concept has been found to be abstract and difficult to understand for some patients (45, 50). At the beginning of our study, the questions about background and identity were often saved for later during the CFI, a choice made by the clinicians. However, when the study had been in progress for a while and the clinicians were more used to the CFI, the questions were asked in the intended order. The order of the questions was adjusted to attain the best possible communication flow. The questions saved for later at the beginning of the study are the ones in previous research found to be uncomfortable or difficult to understand. Saving these questions for later may reflect both clinicians initially not feeling comfortable with asking the questions but also initial difficulties with making the questions understandable. When the clinicians were more experienced in asking the CFI questions the perceived flow of the CFI questions in the intended order was improved. As stated in the DSM-5, the CFI questions are meant to be adapted as deemed suitable. However, further research is needed to evaluate the extent to which the CFI can be adapted in implementation studies.

The questions about background and identity were not documented as often as other CFI questions, perhaps because they were more difficult to make sense of, for both patients and clinicians, in relation to the current problems. The difficulties with the CFI questions about background and identity point to a need for operationalizing them into less abstract ones. From our results, we suggest that asking about a sense of belonging could be a less abstract way of obtaining information about a patient’s cultural identity(ies) and background, and its/their current meanings and perceived impact on health. However, due to differences between patient groups and contexts, there is no single approach, without testing such, to encouraging or gaining information about cultural identity.

#### The CFI contribution to psychiatric diagnostics

Earlier studies have shown that using a culturally sensitive assessment tool in psychiatric assessments can lead to a revaluation of psychiatric diagnoses (28, 39, 60). The RCT from which the analyzed interviews of this study originate showed that the use of the CFI facilitated identification of depression diagnoses and comorbidity among non-native Swedish speaking patients (44). In another study, originating from the same RCT, debriefing instruments for patients and clinicians were used, both designed for the DSM-5 field trial to measure the factors Clinical Utility, Feasibility, and Acceptability (the debriefing instruments for patients slightly modified) (49). Results from the debriefing instrument for clinicians showed positive results on all three factors, which substantiates the perception that the CFI facilitated the psychiatric assessment. In following focus group interviews, the clinicians explained that the information gained through the CFI provided diagnostic clues and helped them identify which aspects of the problem were related to psychiatric illness. Results from the debriefing instrument for patients showed higher scores than those from the debriefing instrument for clinicians. Especially high were the scores on the acceptability question shaped as a VAS scale about the overall perception of the CFI (50).

From this qualitative analysis of the CFI answers from non-native speaking patients, we found that somatic and emotional expressions were often described both in an integrated manner and in relation to the situation in which they arose. The CFI also contributed with rich information about functional disabilities, fatigue, sleeping problems, diminished interest or passivity, and negative impact on cognitive functions and social relations, as well as information concerning resources for daily functioning. These findings could be an explanation of the higher frequency of depression diagnoses and comorbidity among non-native Swedish speaking patients in the RCT, where the CFI was used compared with a control group, and indicates that the CFI questions can contribute to identification of diagnostic categories, especially depression disorders. The information from the CFI indicated signs of psychiatric symptoms corresponding with the DSM-5 criteria for major depression and contributed important pieces of clinical information about distress and decreased functioning, like the inability to be active, concentration problems and sleeping difficulties. The information likely influenced the clinicians’ interpretation of problem descriptions and may have facilitated identification of psychiatric disorder diagnoses, in particular identification of signs of depression. The information given may also have contributed to diagnostic refinement by facilitating identification of other psychiatric symptoms and conditions, denoting identification of comorbidity.

There is lack of research on using the CFI with non-native speakers and when interpreters are used. In clinical situations, psychiatric symptom presentations cannot just be translated between languages without further interpretation of their meaning (61, 62). Not all languages include words directly corresponding to psychiatric diagnostic categories and terminology, for example, to the term of depression (63) or to key diagnostic symptoms of depression, such as depressed mood (62, 64, 65). Symptoms of depression are often expressed in somatic terms instead of psychological ones (19, 25, 26). For identification of depression, more detailed narratives and descriptions of dysfunction can support identifying symptoms of depression in transcultural encounters. The contextualized information of both somatic concerns and emotions revealed during the CFI, as well as information about the patient’s disabilities, may have been helpful in psychiatric diagnostic evaluations, especially contributing to identification of depressive disorders among non-native Swedish speaking patients.

Our results suggest that using the CFI can contribute to increasing a mutual understanding in situations when the patient and clinician do not share a native language, and the rich and contextualized narratives supported by the CFI may facilitate bridging language barriers. As a person-centred method, the CFI may not only strengthen the trust and alliance-building processes in the current therapeutic relationship but also help to repair memories of neither being understood nor respected in prior therapeutic relationships in which no safe space for such patient narration was given.

### Strengths and limitations

A limitation of this study is that the analysis is of the documentation from the CFI answers, and not from the patients’ actual answers. Although the detailed wording of the CFI documentation gives the impression that these are the words of the patients, we cannot be sure what the patients said during the interviews. This means that we have analyzed the clinicians’ interpretations of the patients’ perspective, which is a limitation. However, the clinicians documented the CFI by using the patients’ wordings and perspectives. In our study, an interpreter was used with 11 out of 47 non-native Swedish speaking patients. Although the perception was that an interpreter was not needed in the consultations with the remaining 36 patients, we cannot know how communication was affected when the patients were not able to answer the questions in their own native language. Additionally, we do not know how the use of an interpreter affected communication in the other 11 consultations No sub-group analysis based on gender was done in this study. This is a limitation since we are therefore unable to identify any gender differences in the information gathered by the CFI.

A strength with the present study is the comprehensive data, with information from 47 patients’ answers to the CFI questions documented in detail by many different clinicians. In addition to thematic analysis, the comprehensive dataset enabled us to quantify and account for the frequency of quotes for each sub-theme, providing an added value to the qualitative analysis. It is important to note that the frequency of references is not the point of qualitative analysis, although certain patterns of quantizing responses provide clues of relevance for the diagnostic process (66). The thorough process of overseeing the meaning units several times was an important part of the analytic process and is a methodological strength.

## Conclusion and implications

We have explored what information the CFI questions revealed when used at psychiatric outpatient clinics with 47 newly referred non-native speaking patients. We found that the CFI information contained contextualized descriptions of dysfunction and current life conditions, as well as expressions of emotions, often described along with somatic terms. In this study, the CFI above all invited patients to share, in a narrative form, information related to their daily difficulties, problem descriptions and problem solving. This type of contextual information may facilitate clinicians’ identification of psychiatric symptoms, dysfunctions and disorders, contribute to patients’ feelings of being understood in their current life situation, and also identify possible resources for planning the treatment process.

The results of our study indicate that the narrative approach of the CFI, giving contextualized information about distress and functioning, can facilitate clinicians’ identification of psychiatric symptoms when language, psychiatric terms and understandings are not shared between patient and clinician.

Although we did not analyze the interviews separately for each domain of the CFI, we found that our themes in general focus on the information areas covered in the domains. However, we see a need for development of the questions about culture and identity. For future refinements of the CFI, we suggest that questions about sense of belonging may facilitate eliciting information about patients’ cultural identity(ies) and its/their current meaning and impact on health.

Regarding clinical implementations, our findings point to the value of using the CFI in psychiatric clinical assessments for identifying psychiatric symptoms with non-native speaking patients and facilitating mutual understanding between patient and clinician.

## Data availability statement

The datasets presented in this article are not readily available because of confidentiality of the patients’ medical health records. Requests to access the datasets should be directed to Sofie Bäärnhielm sofie.baarnhielm@regionstockholm.se.

## Ethics statement

The studies involving humans were approved by Regional Ethical Review Board in Stockholm (2015/243-31/2). The studies were conducted in accordance with the local legislation and institutional requirements. The participants provided their oral informed consent to participate in this study.

## Author contributions

MIW: Conceptualization, Data curation, Formal analysis, Methodology, Writing – original draft, Writing – review & editing. VDM: Conceptualization, Supervision, Formal analysis, Validation, Writing – review & editing. LN: Conceptualization, Methodology, Supervision, Writing – review & editing. SB: Conceptualization, Data curation, Formal analysis, Methodology, Supervision, Validation, Writing – review & editing.
